# Effectiveness of Antibacterial Surfaces in Osseointegration of Titanium Dental Implants: A Systematic Review

**DOI:** 10.3390/antibiotics10040360

**Published:** 2021-03-28

**Authors:** Nansi López-Valverde, Bruno Macedo-de-Sousa, Antonio López-Valverde, Juan Manuel Ramírez

**Affiliations:** 1 Department of Surgery, Instituto de Investigación Biomédica de Salamanca (IBSAL), University of Salamanca, 37007 Salamanca, Spain; nlovalher@usal.es; 2 Institute for Occlusion and Orofacial Pain, Faculty of Medicine, University of Coimbra, Polo I-Edifício Central Rua Larga, 3004-504 Coimbra, Portugal; brunomsousa@usal.es; 3 Department of Morphological Sciences, University of Cordoba, Avenida Menéndez Pidal S/N, 14071 Cordoba, Spain; jmramirez@uco.es

**Keywords:** titanium dental implants, antibacterial coating surfaces, osseointegration

## Abstract

Titanium (Ti) dental implant failure as a result of infection has been established at 40%, being regarded as one of the most habitual and untreatable problems. Current research is focused on the design of new surfaces that can generate long-lasting, infection-free osseointegration. The purpose of our study was to assess studies on Ti implants coated with different antibacterial surfaces, assessing their osseointegration. The PubMed, Web of Science and Scopus databases were electronically searched for in vivo studies up to December 2020, selecting six studies that met the inclusion criteria. The quality of the selected studies was assessed using the ARRIVE (Animal Research: Reporting of In Vivo Experiments) criteria and Systematic Review Center for Laboratory animal Experimentation’s (SYRCLE’s) risk of bias tool. Although all the included studies, proved greater osseointegration capacity of the different antibacterial surfaces studied, the methodological quality and experimental models used in some of them make it difficult to draw predictable conclusions. Because of the foregoing, we recommend caution when interpreting the results obtained.

## 1. Introduction

More than 50 years ago, Bränemark described the process of osseointegration as “a direct structural and functional connection between ordered, living bone and the surface of a load-carrying implant”. This researcher proved that an implant’s titanium (Ti) oxide coating could fuse to living bone and that Ti and bone would be impossible to separate without fracturing [1,2].

The process of osseointegration basically consists of an anchoring mechanism through which Ti effectively bonds with the living bone, remaining under all normal load conditions and providing prostheses with long-term clinical stability [3,4]. Despite this, direct bone-to-implant contact could be indicative of a lack of systemic or local response to the implant’s surface and, therefore, osseointegration would involve a biologically negative tissue response [5]. Nevertheless, as reported in earlier publications [6], osseointegration remains a complex and unknown process that depends on certain systems such as the immune system and the autonomic nervous system.

Although postoperative infections after implant surgery are uncommon, some failures are due to infection at the moment of placement or in the following days [7], with a prevalence of around 12% [8]. Moreover, even completely sterilized Ti implants are prone to bacterial infections, sometimes as a result of the host’s defenses being compromised and others due to the questionable antibacterial properties of Ti that have been reported in certain studies [9]. Bacterial infections around implant surrounding tissue (peri-implantitis), whose criteria were established at the World Workshop on the Classification of Periodontal and Peri-implant Diseases and Conditions [10], are one of the most common and untreatable problems associated with Ti dental implants, compromising their integration and destroying their stability, leading to eventual failure [11].

**Figure 1:**
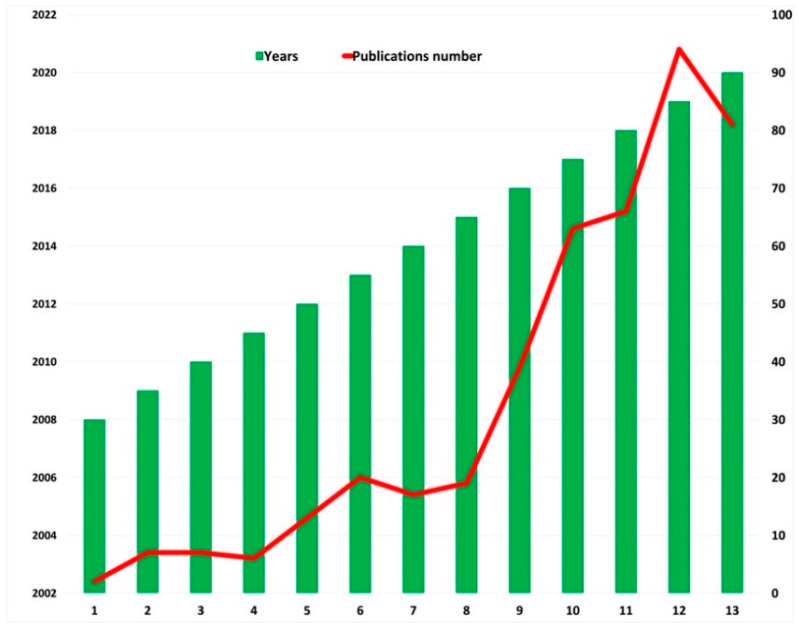
Increase in publications in recent years, with the keywords “Ti dental implants” AND “antibacterial surfaces coating” Source: Web of Science.

This, alongside the need of early osseointegration, is one of the reasons why research in recent decades has focused more on implant surfaces than on the geometry and design of the devices, the aim being to achieve safer and longer osseointegration periods, after the testing and description of different dental implant coatings with antibacterial properties such as different molecules, metals, minerals, antibiotics and antiseptics, among others [12,13,14,15,16,17,18,19]. Figure 1 illustrates the increase in the number of publications that has taken place in recent years.

The purpose of this study was to assess, in vivo, different endosseous Ti devices coated with a variety of antimicrobial agents aimed at enhancing osseointegration.

## 2. Results

### 2.1. Search Results and Study Description

**Figure 2:**
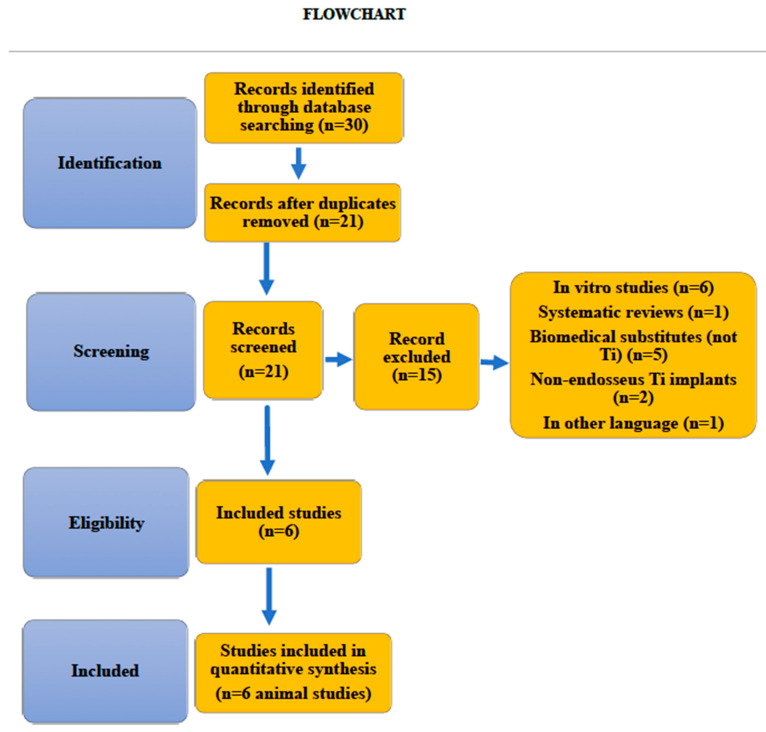
Flowchart.

Until December 2020, a total of 30 studies were selected and independently assessed by two reviewers. A total of 6 studies were included in the systematic review (Figure 2 Flowchart). 212 implants coated with 6 different antibacterial surfaces were assessed. No meta-analysis was conducted because of the scarcity and heterogeneity of the studies.

### 2.2. Description of the Characteristics of the Studies

Table 1, Table 2 and Table 3 provide a general description of the characteristics of the included studies. The studies used 4 experimental models (rabbit [20], mouse [21], rat [22] and dog [19,23,24]). Sample sizes varied between 3 [19] and 36 animals [22]. Two studies [19,20] used metal coatings (Ta, Sr), two used antibiotic coatings (DC, BC) and two used [23,24] rhBMP. The longest monitoring period was 8 weeks [20,21,23,24]. All the studies reported on bone formation around implants, the most used measurement method being BIC [20,21,22,23,24]. Only two of the studies assessed antibacterial activity of coating surfaces [19,22].

### 2.3. Risk of Bias and Quality Assessment of the Studies Included

**Figure 4:**
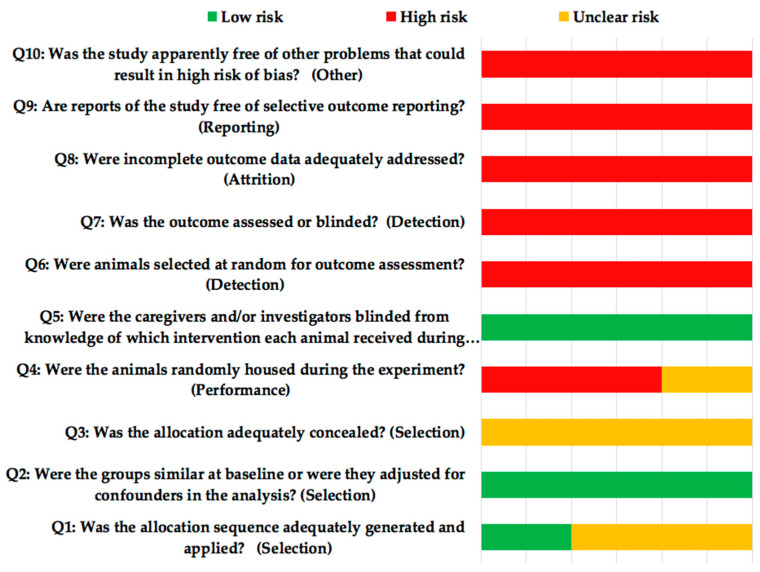
SYRCLE’s (Systematic Review Centre for Laboratory Animal Experimentation) risk of bias tool.

Risk of bias was assessed according to the SYRCLE guide (Systematic Review Center for Laboratory animal Experimentation) [25] (Figure 4). All the studies presented high risk of bias. The quality of the selected studies (ARRIVE [Animal Research: Reporting of In Vivo Experiments] criteria, Table 4) achieved a mean score of 17.6. None of the studies reported items 5 (Reasons for animal models), 19 (3Rs, Replace, Reduce and Refine), 20 (Adverse events) and 21 (Study limitations), although Susin et al. [24] made reference in the discussion of their article to the beginning of the 3Rs, without mentioning if they complied with it. Only one of the studies [21] failed to report item 22 (Generalization/Applicability).

## 3. Discussion

Dental implant surfaces are under constant research and evolution. Despite reporting survival rates above 95% [26], traditional SLA surfaces (Sandblasted, Large-Grit, Acid-Etched Surface), are not free from disadvantages, one of them being hydrophobicity, which has led to additional modifications of this type of surface [27,28]. Another issue is the time required for bone healing which, although it has considerably reduced, still involves a lengthy period [29].

In particular, oral cavity conditions (abundance of fluoride ions, lactic acid and certain microorganisms), resistance to corrosion and the antibacterial properties of Ti diminish, which could lead to premature surface infections and eventual implant failure [30,31]. Likewise, although the rough surfaces that currently characterize most dental surfaces favor osseointegration, certain authors have reported that such surfaces have the disadvantage of also favoring infection of the tissues that surround the implant (peri-implantitis) [32]. Like osteogenic cells, oral cavity bacteria have an affinity for rough Ti surfaces, causing a true race to colonize its surface [33,34]. After colonization, the host develops an inflammatory response, generating proinflammatory cytokines that stimulate the genesis of osteoclasts and increase the risk of peri-implantitis [35,36], whose prevalence stands at up to 40%, depending on the site [37,38].

Of the 6 studies selected for our review, two [19,20] used metal coatings (Ta, Sr) with antimicrobial properties on Ti surfaces, two [21,22] used antibiotics coatings (DC, BC) and two [23,24] used human bone morphogenetic protein (rhBMP).

Traditionally, the metallic compound that has been most frequently used as antibacterial has been silver (Ag) [39], followed by others such as Ta, Sr, Zinc (Zn), Ti and Copper (Cu). Certain researchers have studied Ta’s biocompatibility and corrosion resistance [40], proving that porous Ta might allow bone formation and favor not only osseointegration, but also osseoincorporation, which would significantly improve the secondary stability of implants in bone tissue. On this aspect, in a study on Beagle dog models, Zhang and colleagues [19] used Ta coatings on Ti implants, reporting greater antibacterial capacity and greater osseointegration.

Sr salt (Sr ranelate, SrRan) has been clinically used to treat osteoporosis, even though its mechanism of action on bone remodeling remains unknown. There is in vitro evidence of SrRan acting on mesenchymal cells in their osteogenic differentiation [41], reducing the attachment of osteoclasts to the bone surface by increasing collagen synthesis and alkaline phosphatase [42], thus improving osseointegration and early implant binding [43,44]. Zhou and colleagues [20] proved, in vitro and in vivo, that the addition of Sr to Ti oxide surfaces (TiO_2_) improved their osteogenic capacity as well as enhancing antigenic and antibacterial activity; however, they realized that altering such surfaces with high contents of Sr would deteriorate such capacities. Nevertheless, in their systematic reviews, Shi and colleagues and López-Valverde and colleagues [45,46] reported differences in bone formation around Ti implants coated with Sr, depending on the experimental model, considering it optimal in rat models and non-significant in other models such as rabbits. Such differences were attributed to possible dynamic bone formation and remodeling differences, especially in early healing intervals. A large number of in vitro and in vivo studies have revealed that, as well as good cytocompatibility, the addition of Sr and Ag to TiO_2_ surfaces encourages strong antibacterial activity and accelerates new bone formation around the implant [47,48,49,50].

Microarc Oxidation (MAO) or Plasma Electrolitic Oxidation (PEO) is an electrochemical treatment that results in a more stable oxide layer than anodic oxidation. If the electrolyte in which PEO is performed contains calcium and phosphate ions, the oxide layer produced may contain HA. This ceramic layer possesses high stability and resistance to corrosion and wear, enhancing the host cellular reaction in terms of osteoblastic proliferation and differentiation, considered one of the most promising techniques, due to the formation of a high bond between the bone and the surface of Ti [51,52,53,54,55]. In our review, the study by Ding and colleagues [21] evaluated in mice, the formation of new bone on HA (cathodic sputtering) coated implant surfaces treated with DC in an oral environment, concluding that this coating would promote bone apposition around the implant. However, HA has been used as a vehicle for antibiotic delivery because commercial HA itself shows no activity against Gram-positive and Gram-negative bacteria [56,57]. Nevertheless, some studies have pointed out the need to evaluate the biocompatibility and tissue integration capacity of PEO-coated surfaces, as well as their corrosion resistance and antibacterial capacity in vivo [58,59,60,61,62].

The use of antibiotic coatings (such as bacitracin, amoxicillin, doxycycline, gentamycin) on dental implant surfaces could chemically improve molecular and cellular responses and reduce infection rates, facilitating osseointegration [63,64]. As well as acting as a potential factor in the treatment of periodontal diseases, doxycycline is one of the antibiotics that are commonly used to control infection after implant surgery [65,66]; therefore, incorporating this drug into implant surfaces could control the speed of release on the implant site [67]. In an in vivo study using mice, Ding and colleagues [21] reported a significant increase in BIC at 4 and 8 weeks in the doxycycline-coated implant group as compared with the HA-coated implants. Their results are consistent with those of other studies that propose doxycycline as an ideal bacteriostat that would remain on the implant’s surface for at least two weeks following implant placement, without altering surface topography [65,68,69]. In a study using rat femurs, Nie and colleagues [22] found significant differences between implants that had been altered with BC and Ti implants in implant sites that were contaminated with *Staphylococcus aureus* bacteria, reporting better osteogenic capacity in the BC-coated implants and therefore concluding that Ti–BC implants could promote bone formation. In a previous in vitro study, these same authors had proved the capacity of immobilized BC to enhance Ti hydrophilicity and that titanium immobilized with BC could inhibit bacterial attachment and colonization [70]. Nevertheless, certain studies have reported the problems that antibiotic coated implants could generate, among which are loss of bactericidal capacity and the generation of antibiotic-resistant strains [71].

The last two studies included in our review, those by Lee and colleagues and Susin and colleagues [23,24], used Hound Labrador Mongrel dogs to assess new bone formation around endosseous Ti implants totally or partially coated with rhBMP-2 and rhBMP-7 (Human Bone Morphogenetic Protein), placed in critical size bilateral peri-implant supra-alveolar defects. With their different subtypes, BMPs (bone morphogenetic protein) are the most powerful osseoinducers known to date [72]. BMP-2 plays an essential role in chondrogenesis, osteogenesis and revascularization processes, anticipating that the other BMPs are incapable of replacing the function of BMP-2 in bone healing [73]. Cohen and colleagues [74] demonstrated in vitro that this protein would act as an immunomodulator in bacteria-infected neutrophils. Likewise, the presence of rhBMP-2 on the implant bed would stimulate and activate the infiltrating neutrophils that are the first line of defense in acute inflammatory response. They also reported that the production of reactive oxidative species on contaminated surgical sites would indicate the role of rhBMP-2 as a priming agent for neutrophils, increasing their bactericidal capacities. Certain clinical trials have shown how low concentrations of certain bactericidal agents combined with BMP-2 were able to almost completely suppress bacterial growth as compared to treatments that did not use BMP-2.

Among other functions, BMP-7 plays a role in healing and regenerating the skeleton, being regarded as an important mediator in osteoblastic differentiation as well as a powerful anti-inflammatory and antioxidant [75,76,77]. Susin and colleagues [24] reported that coating porous Ti oxide surfaces with rhBMP-7 would stimulate bone formation, enhancing osseointegration and vertical growth of the alveolar crest; nevertheless, they warned that the use of high concentrations of rhBMP-7, could give rise to local side effects.

Lee and colleagues [23] reported that full coating of the implant surface with rhBMP-2 would favor osseointegration and bone remodeling in compromised bones (type IV according to Lekholm and Zarb [78]) and that local application of rhBMP-2 on the most coronal part of the implant would provide an ideal coating to extrapolate animal studies to clinical trials (RCTs). In this regard, Chen ad colleagues, Ji and colleagues and Helbig and colleagues researched the effects on osteogenesis on chronically infected sites, proving that these proteins were capable of maintaining osteoinductivity in the presence of infection [79,80,81,82].

Direct coating of implant surfaces using BMP has been assessed in preclinical models with promising results as a feasible alternative to current bone augmentation procedures [83]. The use of rhBMP-7 as an alternative to autologous bone grafts has been approved both in Europe and in the USA, there being numerous studies that promote its use in the treatment of certain types of pseudoarthrosis [84,85]. There is proof that this protein plays an important role in M2 macrophage and monocyte polarization and is decisive to increase the expression of anti-inflammatory cytokines [86,87], since it is known that the presence of monocytes/macrophages in the early stages and their transition to multinucleated cells, coincides with the formation of ectopic bone around biomaterials [88]. Ultimately, Ti substrates that release antibiotics and osteoinductive proteins (BMPs) would improve the function of osteoblastic cells and could be a promising material to promote osseointegration and longevity of implants in orthopedics and dentistry [89,90,91].

However, our systematic review has a series of limitations: first and foremost, the small number of studies, which precludes meta-analysis; second, there is significant variation in cortical bone formation and remodeling among the different experimental models, added to the fact that implant sites in some of the included studies (rat femurs) are not adequate models to extrapolate results to humans; third, the quality and methodology of the included studies proved very disparate, hindering result comparison; fourth, preclinical studies always provide less evidence and applicability to patients than clinical trials, since they evaluate the effect of an intervention in cell or animal models.

Although the six studies included reported positive effects as regards the effectivity of the antimicrobial coatings used, we believe that, in order to determine the efficacy of a certain surface, it is necessary to reduce biases, establish appropriate research parameters and eliminate confounders, the purpose of this being to obtain useful and clinically applicable results.

## 4. Materials and Methods

### 4.1. Protocol and Register

This study was designed by NL-V. The review was performed according to the PRISMA guidelines for systematic reviews [92] (Table S1, Checklist), using a specific question based on the PICO framework:

(P) Participants: subjects who received endosseous implants.

(I) Intervention: modified implants coated with antibacterial surfaces.

(C) Control: non-modified Ti implants.

(O) Outcome: soft tissue response and bone formation around modified Ti implants—BIC (bone implant contact), BA (bone area) and BD (bone density).

The research question was: “Are antibacterial-doped titanium surfaces more osseointegrative than etched surfaces (SLA)?”.

### 4.2. Selection Criteria, Information Sources and Search

Exclusion criteria: Studies that did not use Ti surfaces coated with antibacterial surfaces or did not evaluate antibacterial activity; studies on modified animals (osteoporotic, diabetic…); in vitro studies; narrative reviews and systematic reviews, and studies published in languages other than English.

The PubMed, Web of Science (WOS) and Scopus databases were searched for articles published until December 2020. The MeSH terms (Medical Subject Headings) used in the PubMed databases were: “Dental Implants” [MeSH Terms] AND “Coated Materials” [MeSH Terms] AND “Biocompatible” [MeSH Terms] AND “Anti-Bacterial Agents” [MeSH Terms] AND “Animals” [MeSH Terms] AND “Osseointegration” [MeSH Terms]. The Boolean operator AND was used to refine the search.

### 4.3. Data Extraction and Analysis

The titles and abstracts of the articles yielded by the three search engines (PubMed, WOS and Scopus) were downloaded using Mendeley software (Elsevier Inc, New York, NY, USA, EE. UU.). Two reviewers (NL-V and AL-V) independently selected the titles and abstracts, and disagreements regarding inclusion were settled through discussion. The full texts of the selected articles were obtained and reviewed for inclusion.

### 4.4. Risk of Bias

This was assessed using an adapted version of the Cochrane RoB tool (Risk of Bias Tool), with specific biases for animal studies (SYRCLE’s RoB, Systematic Review Centre for Laboratory Animal Experimentation) [25].

### 4.5. Quality of the Selected Articles

This was assessed using the modified ARRIVE guidelines (Animal Research: Reporting of In Vivo Experiments) with 23 items that were rated by the two mentioned reviewers (NL-V, AL-V), with scores of 0 (not reported) or 1 (reported) [93]. (Table 4. ARRIVE guidelines).

## 5. Conclusions

According to all the assessed preclinical studies, implants with antibacterial coatings proved greater osseointegration than control surfaces; nevertheless, because of the limitations of our review, it is difficult to conclude that such surfaces might have greater osseointegration capacity, mainly because all the studies were biased in important methodological aspects. Therefore, the conclusions arrived at must be taken with relative caution.

The main approach in the development of any type of implant consists of minimizing bacterial adhesion during the proliferation of osteogenic and fibroblastic cells, with the purpose of achieving high levels of hard and soft tissue integration. This requires the development of multifunctional surface coatings. Hence, future research should focus on the design of a single type of multipurpose implant with improved clinical behavior regarding bone and fibrous integration and which may, in turn, prevent infections of implant surrounding tissues.

## Figures and Tables

**Figure 3:**
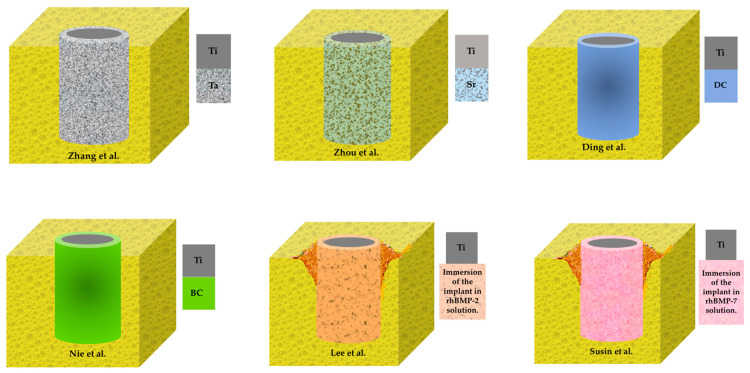
Graph of the incorporation of antibacterial surfaces to Ti-implants in included studies. Ti, titanium; Ta, tantalum; Sr, strontium; DC, doxycycline; BC, bacitracin; rhBMP, human bone morphogenetic protein.

**Table 1:** Characteristics of included studies.

Studies	Animal Model (n)	Location of Implant Placement	Follow-Up	Analysis Methods	Conclusions
Zhang et al. [19]	Beagle dog model (3)	Mandible (premolars and molar area)	4 weeks	- Micro-CT. - Bone volume (BV). - Bone Mineral Density (BMD). - Trabecular Thickness (Tb.Th). - Trabecular Number (Tb.N).	The SLA-Ta (Tantalum) surface showed excellent antibacterial activity against *Porphyromonas gingivalis* and Fusobacterium nucleatum involved in peri- implant infections.
Zhou et al. [20]	New Zealand rabbit model (24)	Femoral shafts area	8 weeks	- Histological analysis of the BIC. - Pull-out force of the metallic Ti wires with and without coatings. - Bacterial counting method	The incorporation of Strontium (Sr) induces a better osseointegration, but it did not affect its angiogenic and antibacterial capabilities.
Ding et al. [21]	Wild mice model (20)	The upper first right molar area	8 weeks	- Micro-CT - BIC - Bone Area (BA)	The doxycycline (DC)-treated Hydroxyapatite (HA)-coated implant surface promotes bone apposition around the implant.
Nie et al. [22]	Rat model (36)	Femur	3 weeks	- *Staphylococcus aureus* concentration. - Micro-CT. - BIC.	The bacitracin (BC) on the Ti surface demonstrated potential prophylaxis against Ti implant-associated infection. Further, the BC-coated Ti showed potential towards osteoinductvity in a rat model.
Lee et al. [23]	Hound Labrador dogs (12)	Mandibular premolar area	8 weeks	- Radiographic recordings (Presence of a periimplant radiolucent zone). - BIC - BMD	Human bone morphogenetic protein- 2 (rhBMP-2)-coated tita- nium porous oxide implants induce significant bone formation.
Susin et al. [24]	Hound Labrador dogs (6)	Mandibular premolar area	8 weeks	- Radiographic recordings (Presence of a periimplant radiolucent zone). - BIC - BMD	rhBMP-7 coated onto Ti porous-oxide surface implants induces clinically relevant local bone formation including osseointegration and vertical augmentation of the alveolar ridge.

BV, bone volume; BMD, bone mineral density; Tb.Th, trabecular thickness; Tb.N, trabecular number; BIC, bone to implant contact; Ti, titanium; Ta, tantalum; Sr, strontium; BA, bone area; DC, doxycycline; BC, bacitracin; HA, hydroxyapatite; rhBMP, human bone morphogenetic protein.

**Table 2:** Characteristics of implants.

Studies	Implants Number (n)	Implant Dimensions, D(Ø) × L (mm)	Ti Implant Shape	Antibacterial Surface Incorporation (See Figure 3)	Surface Coating
Zhang et al. [19]	24	3.3 Ø × L 10	Screw	Tantalum (Ta)	The Ti base was sputtered Ti sprayed for 10 min. Then, Ta deposition was carried out for 40 min by sputtering.
Zhou et al. [20]	24	2.5 Ø × L 10	Cylinder	Strontium (Sr)	The adhesion force and ion release of the coating are shown in figure.
Ding et al. [21]	20	0.8 Ø × L 1.5	Screw	Doxycycline (DC)	Frequency sputtering method.
Nie et al. [22]	36	1.5 Ø ×L 20	Rod	Bacitracin (BC)	BC grafted on the surface of Ti bacitracin (concentration 1 mg/mL).
Lee et al. [23]	72	4.3 Ø × 10 L	Screw	30 µg rhBMPm-2/implant was applied.	Immersion of the entire implant in an rhBMP-2 solution.
Susin et al. [24]	36	4 Ø × 10 L	Screw	30 µg rhBMPm-7/implant was applied.	Immersion of the entire implant in an rhBMP-7 solution.

Ta, tantalum; Sr, strontium; DC, doxycycline; BC, bacitracin; rhBMP, human bone morphogenetic protein.

**Table 3:** Antibacterial activity and bone formation in vivo. Outcomes.

Studies, Year	Antibacterial Activity	Bone Formation
Zhang et al. [19]	The SLA-Ta surface hampered the biofilm formation of *P. gingivalis*, although the mechanism of antibacterial activity of the SLA-Ta surface remains unknown.	Better osseointegration of the Ta coating. The BIC and BD of the coated implants (SLA-Ta) was significantly higher than that of those not modified with Ta (*p* < 0.05).
Zhou et al. [20]	NR	The Sr coatings gave the implants better osseointegration ability compared to bare metal Ti substrates. BIC *p* < 0.01 compared to metallic Ti substrate.
Ding et al. [21]	NR	At 4 and 8 weeks, BIC of DC group, was significantly higher than the one of HA group.
Nie et al. [22]	The number of bacteria in the bacitracin (BC) modified Ti implant was significantly lower compared to the unmodified Ti rod group.	BIC for the Ti–BC implants were significantly higher than those of the Ti-implants (*p* < 0.05).
Lee et al. [23]	NR	- The induced bone was thin trabecular bone, with restricted BIC. - Lamellar bone formation in at implants with to localized rhBMP-2 coating. - BD averaged 38.0 ± 3.8% and 34.4 ± 5.6% - for coronal- and soak-load implants, - respectively (*p* > 0.05). - BIC-values averaged 25.0 ± 3.8% - and 31.2 ± 3.3% (*p* > 0.05).
Susin et al. [24]	NR	BIC values for Ti implants versus Ti coated with rhBMP-7 44 ± 17 and 40 ± 9%, respectively. BD values were 44 ± 17% versus 40 ± 9%, respectively.

NR, not reported; BIC, bone implant contact; BD, bone density; Ta, tantalum; Sr, strontium; DC, doxycycline; BC, bacitracin; HA, hydroxyapatite.

**Table 4:** Checklist of ARRIVE criteria reported by the included studies.

Studies	Zhang et al. [19]	Zhou et al. [20]	Ding et al. [21]	Nie et al. [22]	Lee et al. [23]	Susin et al. [24]
1. Title	1	1	1	1	1	1
Abstract						
2. Species	1	1	1	1	1	1
3. Key finding	1	1	1	1	1	1
Introduction						
4. Background	1	1	1	1	1	1
5. Reasons for animal models	0	0	0	0	0	0
6. Objectives	1	1	1	1	1	1
Methods						
7. Ethical statement	1	1	1	1	1	1
8. Study design	1	1	1	1	1	1
9. Experimental procedures	1	1	1	1	1	1
10. Experimental animals	1	1	1	1	1	1
11. Accommodation and handling of animals	0	1	0	1	0	0
12. Sample size	1	1	1	1	1	1
13. Assignment of animals to experimental groups	1	1	1	1	1	1
14. Anesthesia	1	1	1	1	1	1
15. Statistical methods	1	1	1	1	1	1
Results						
16. Experimental results	1	1	1	1	1	1
17. Results and estimation	1	1	1	1	1	1
Discussion						
18. Interpretation and scientific implications	1	1	1	1	1	1
19. 3Rs reported	0	0	0	0	0	0
20. Adverse events	0	0	0	0	0	0
21. Study limitations	0	0	0	0	0	0
22. Generalization/ applicability	1	1	0	1	1	1
23. Funding	0	0	0	1	1	1
TOTAL, SCORE	17	18	16	19	18	18

17.6 ± 1.03. Score and mean deviation of the studies included.

## Data Availability

Not applicable.

## Supplementary Materials

The following are available online at https://www.mdpi.com/article/10.3390/antibiotics10040360/s1, Table S1: PRISMA Checklist.

## Abbreviations

Ti	Titanium
Ta	Tantalum
Sr	Strontium
BIC	Bone to Implant Contact
BA	Bone Area
BD	Bone Density
MeSH	Medical Subject Headings
HA	Hydroxyapatite
BC	Bacitracine
DC	Doxycycline
rhBMP	Human Bone Morphogenetic Protein
BV	Bone Volume
BMD	Bone Mineral Density
Tb.Th	Trabecular Thickness
Tb.N	Trabecular Number
